# Supplementary figures and images for: Morphological characterization of virus-like particles in coral reef sponges
Source: PeerJ. 2018 Oct 17;6:e5625. doi: 10.7717/peerj.5625 (PMC6195793; doi:10.7717/peerj.5625)

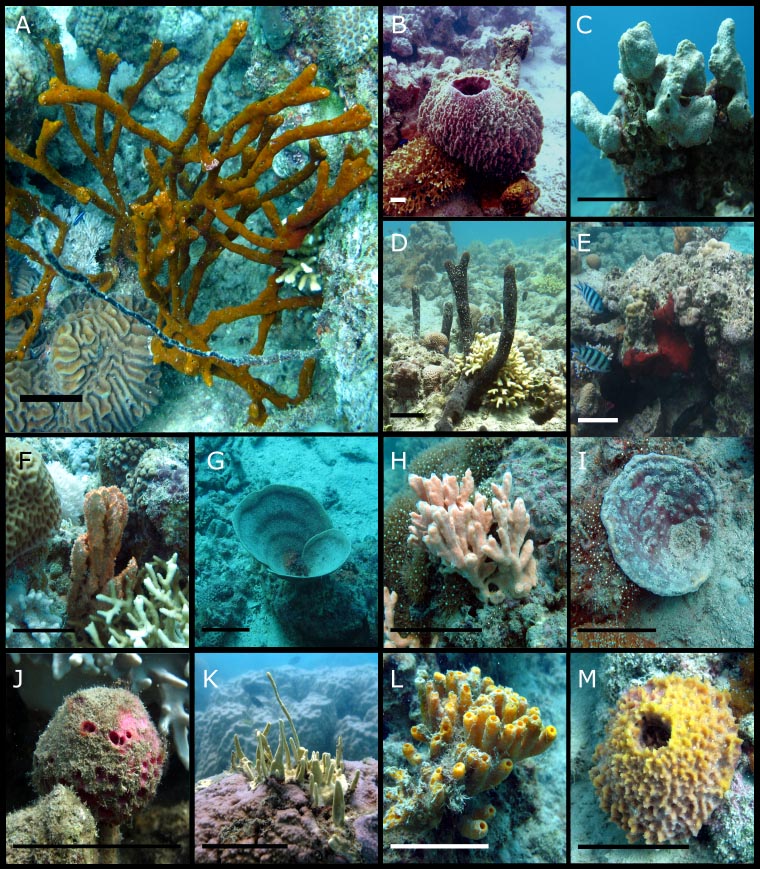

Supplement: Figure S1 — Red Sea sponge species: (A) Amphimedon ochracea, (B) Xestospongia testudinaria, (C) Crella cyathophora, (D) Hyrtios erectus, (E) Mycale sp. GBR and Red Sea sponge species: (F) Stylissa carteri, (G) Carteriospongia foliascens. GBR sponge species: (H) Echinochalina isaaci, (I) Cymbastella marshae, (J), Cinachyrella schulzei, (K), Lamellodysidea herbacea, (L), Pipestela candelabra, (M) Xestospongia sp.. Scale bar = 10 cm. Photos by Cecília Pascelli. [file peerj-06-5625-s001.jpg]
