# Supplementary material for: Morphological characterization of virus-like particles in coral reef sponges
Source: PeerJ. 2018 Oct 17;6:e5625. doi: 10.7717/peerj.5625 (PMC6195793; doi:10.7717/peerj.5625)
Supplement: Table S1 [file peerj-06-5625-s002.docx]

| VLP Morphotype | Site | Associated sponge specie | Classification | Size range | Details |
| --- | --- | --- | --- | --- | --- |
| M-I | GBR | *Carteriospongia foliascens* | Tailed (short) non-enveloped, spherical-shaped VLP (icosahedral symmetry), with electron-dense core. | 210 nm in diameter, core with 194 nm in diameter, tail with 25 nm long and wide. | Observed in the cellular lumen of sponge archaeocytes. The icosahedral structure and the short tail are characteristic of the *Podoviridae*. |
| M-II | GBR | *Xestospongia* sp. | Tailed (short), non-enveloped VLP, with icosahedral symmetry. | Capsid with 52 nm diameter, core with 40 nm in diameter. Tail with 48/21 nm long/wide. | Observed in sponge mucus. |
| M-III | GBR | *Echinochalina isaaci* | Tailed (short), non-enveloped VLP, with icosahedral symmetry. | Head measuring 50 nm in diameter. Tail measuring 14 nm long, 11 nm wide. | Observed in sponge mucus. |
| M-IV | RS | *Stylissa carteri* | Tailed (short), non-enveloped VLP, with icosahedral symmetry. | Capsid with 75-78 nm diameter and tail with 24-25 nm long/wide. | Observed in sponge mucus and characteristic of Podoviridae family |
| M-V | GBR | *Cinachyrella schulzei* | Non-enveloped tailed bacteriophage with symmetric icosahedral head. | 150 nm in total length, with symmetric hexagonal head measuring 55 nm in diameter and an elongated tail measuring 95 nm in length and 10 nm in width. | Observed in sponge mucus. |
| M-VI | GBR | *Echinochalina isaaci* | Non-enveloped tailed bacteriophage with symmetric icosahedral head. | 260 nm in total length, with symmetric hexagonal head measuring 90 nm in diameter and an elongated tail measuring 160 nm in length and 1nm in width. | Observed in sponge mucus. |
| M-VII | GBR | *Echinochalina isaaci* | Filamentous VLP with electron-dense core. | 200 nm in total length, with elongated head measuring 95 nm in length and 70 nm in width and an elongated tail measuring 105 nm in length and 22.5 nm in width. | Observed in sponge mucus. |
| M-VIII | RS | *Stylissa carteri* | Non-enveloped T4-like bacteriophage. | 283 nm in total length, with elongated hexagonal head measuring 128 nm in length and 84 nm in width and tail measuring 155 nm in length and 26,6 nm in width. | Observed in sponge mucus and strong resemblance to Myoviridae. |
| M-IX | RS | *Amphimedon ochracea* | Non-enveloped tailed bacteriophage with symmetric icosahedral head. | 142 nm long, head measuring 59.8 nm in diameter with electron-dense core with 36.4 nm in diameter. Tail measuring 82 nm long, 12 nm wide. | Observed in the sponge mucus. The VLP features are characteristic of Siphoviridae. |
| M-X | GBR | *Carteriospongia foliascens* | Non-enveloped, icosahedral symmetry VLP, with electron-dense core. | 150 nm in diameter; core with 122 nm in diameter. | Observed in sponge mesohyl. |
| M-XI | GBR | *Carteriospongia foliascens* | Non-enveloped, icosahedral symmetry VLP, with electron-dense core. | 140-150 nm in diameter, core with 90-100 nm in diameter. | Observed in sponge mesohyl and the cellular lumen of sponge archaeocytes. |
| M-XII | GBR | *Carteriospongia foliascens* | Non-enveloped VLP with spherical/icosahedral symmetry and electron-dense core. | 103 nm in diameter, core with 81 nm in diameter. | Observed in sponge mucus. |
| M-XIII | GBR | *Carteriospongia foliascens* | Icosahedral symmetry VLP, with electron-dense core. | 56 nm in diameter, core with 41 nm in diameter. | Observed in sponge mucus. |
| M-XIV | GBR | *Stylissa carteri* | Enveloped VLP with spherical/icosahedral symmetry. | 90-100 nm of diameter. | Purified from sponge tissue via density gradient ultracentrifugation. |
| M-XV | GBR | *Stylissa carteri* | Icosahedral symmetry VLP, with electron-dense core. | 89 +- 4 nm in diameter, core with 74 nm +- 7 nm in diameter. | Observed in sponge mucus. |
| M-XVI | GBR | *Xestospongia* sp. | Non-enveloped, icosahedral symmetry VLP, with electron-dense core. | 70 nm in diameter, core with 37 nm in diameter. | Purified from sponge tissue via density gradient ultracentrifugation. |
| M-XVII | GBR | *Pipestela candelabra* | Enveloped VLP with spherical/icosahedral symmetry. | 180-205 nm in diameter. | Purified from sponge tissue via density gradient ultracentrifugation. |
| M-XVIII | GBR | *Pipestela candelabra* | Non-enveloped VLP with spherical/icosahedral symmetry and electron-dense core. | 138 nm in diameter, core with 35 nm in diameter. | Purified from sponge tissue via density gradient ultracentrifugation. |
| M-XIX | GBR | *Pipestela candelabra* | Non-enveloped VLP with spherical/icosahedral symmetry and electron-dense core. | 180-200 nm in diameter, core with 120-124 nm in diameter. | Observed within gram-negative bacteria. |
| M-XX | GBR | *Pipestela candelabra* | Non-enveloped VLP with spherical/icosahedral symmetry and electron-dense core. | 100 nm in diameter, core with 69 nm in diameter. | Observed in sponge mucus. |
| M-XXI | GBR | *Lamellodysidea herbacea* | Geminate VLP. | 135 nm in diameter. Comprise 2 quasi-isometric particles with 72 nm in diameter each with a dese core with 57 nm in diameter. | Observed in sponge mucus. |
| M-XXII | GBR | *Lamellodysidea herbacea* | Non-enveloped VLP with spherical/icosahedral symmetry and electron-dense core. | 118 nm in diameter, core with 91 nm in diameter. | Observed in sponge mucus. |
| M-XXIII | GBR | *Lamellodysidea herbacea* | Non-enveloped VLP with spherical symmetry and electron-dense core. | 111 nm in diameter, core with 81 nm in diameter. | Observed in sponge mucus. |
| M-XXIV | GBR | *Cinachyrella schulzei* | Non-enveloped VLP with spherical/icosahedral symmetry and electron-dense core. | 70-85 nm in diameter, core with 35-45 nm in diameter. | Purified from sponge tissue via density gradient ultracentrifugation. |
| M-XXV | GBR | *Cinachyrella schulzei* | Non-enveloped electron-dense VLP with icosahedral symmetry. | 135 nm in diameter, core with 95 nm in diameter. | Observed in sponge mucus. |
| M-XXVI | GBR | *Cymbastella marshae* | Non-enveloped, icosahedral symmetry VLP, with electron-dense core. | 74 nm in diameter, core with 42 nm in diameter. | Observed in sponge mucus. |
| M-XXVII | RS | *Carteriospongia foliascens* | Non-enveloped electron-dense VLP with icosahedral symmetry. | 60-65 nm in diameter. core with 32-35 nm in diameter. | Observed within Gram-negative bacteria. |
| M-XXVIII | RS | *Carteriospongia foliascens* | Non-enveloped VLP with icosahedral symmetry and electron-dense core. | 100-110 nm in diameter. | Observed in sponge mesohyl and inside a lysed cyanobacterial cell. |
| M-XXIX | RS | *Stylissa carteri* | Non-enveloped VLP with spherical/icosahedral symmetry and electron-dense core. | 81 nm in diameter, core of 56 nm diameter. | Observed in sponge mucus. |
| M-XXX | RS | *Stylissa carteri* | Non-enveloped VLP with spherical/icosahedral symmetry. | 163-175 nm in diameter. | Observed in sponge mucus. |
| M-XXXI | RS | *Stylissa carteri* | Non-enveloped VLP with icosahedral symmetry. | 60 nm in diameter, core of 42 nm diameter. | Observed in sponge mucus. |
| M-XXXII | RS | *Xestospongia testudinaria* | Non-enveloped VLP with icosahedral symmetry and electron-dense core. | 140-150 nm in diameter, core of 120-125 nm diameter. | Observed in the sponge mesohyl. |
| M-XXXIII | RS | *Hyrtios erectus* | Non-enveloped VLP with icosahedral symmetry and electron-dense core. | 85 nm in diameter, core with 53 nm in diameter. | Single VLP observed inside sponge cell. |
| M-XXXIV | RS | *Hyrtios erectus* | Enveloped VLP with icosahedral symmetry and electron-dense core. | 110-128 nm in diameter, ovoid-shaped core with 50-80 nm in diameter. | Observed within vacuole in the sponge mesohyle matrix and sitting on top of the sponge cell. |
| M-XXXV | RS | *Mycale* sp. | Non-enveloped VLP with icosahedral symmetry and electron-dense core. | 71-85 nm in diameter, core with 54-70 nm in diameter. | Observed in sponge mucus. |
| M-XXXVI | GBR | *Carteriospongia foliascens* | Ovoid-shaped VLP with electron-dense core. | 118 nm long, 85 nm wide, core 82 nm long, 59 nm wide. | Observed in sponge mucus. |
| M-XXXVII | GBR | *Xestospongia* sp. | Rod-shaped Filamentous VLP | 120-130 nm long, 18 nm wide. | Purified from sponge tissue via density gradient ultracentrifugation. |
| M-XXXVIII | GBR | *Xestospongia* sp. | Rod-shaped Filamentous VLP | 171 nm long, 28 nm wide. Core with157 nm long, 23 nm wide. | Observed in sponge mucus. |
| M-XXXIX | GBR | *Cinachyrella schulzei* | Filamentous VLP with electron-dense core. | 400 nm in length and 25 nm in width. With an electrodense core measuring 7.5 nm in diameter. | Observed in sponge mucus. |
| M-XL | RS | *Carteriospongia foliascens* | Filamentous VLP, non-electron-dense core. | 100-130 nm long, 50-60 nm wide | Observed sitting on top of the Cyanobacteria and in the sponge mesohyl. |
| M-XLI | RS | *Stylissa carteri* | Filamentous VLP with electron-translucent core. | 520-600 nm long 12-15 nm wide. | Observed in sponge mucus. |
| M-XLII | RS | *Stylissa carteri* | Rod-shaped filamentous VLP with electron-translucent core. | 230 nm long, 19 nm wide. | Observed in sponge mucus. |
| M-XLIII | RS | *Xestospongia testudinaria* | Filamentous VLP with electron-translucent core. | 340-1300 nm long, 15-30 nm wide. | Observed in sponge mesohyl and in sponge archaeocytes. |
| M-XLIV | RS | *Crella cyathophora* | Filamentous VLP. | 150-154 nm long, 22-25 nm wide. | Observed in the sponge mucus. This VLP tube-like structure resembles Paramyxovridae morphology. |
| M-XLV | RS | *Crella cyathophora* | Non-enveloped VLP with geminate icosahedral capsid and an electron-dense core. | 221 nm long, formed of two hexagonally symmetric portions with 110 nm in diameter. Core measuring 83-90 nm in diameter. | Observed inside a vacuole in the sponge mesohyl. |
| M-XLVI | GBR | *Lamellodysidea herbacea* | Geminate VLP. | 135 nm in diameter. Comprise 2 quasi-isometric particles with 72 nm in diameter each with a dese core with 57 nm in diameter. | Observed in sponge mucus. |
| M-XLVII | RS | *Amphimedon ochracea* | Geminate VLP. | 81-95 nm long, 37-48 nm wide. Comprise 2 quasi-isometric particles with 34-45 nm long each. | Observed in the sponge mucus as well as in association with filamentous Cyanobacteria. VLP was observed inside cell vacuoles, between thylakoid membranes and around stellar bodies. |
| M-XLVIII | RS | *Crella cyathophora* | Enveloped brick-shaped VLP with electron-dense lateral bodies. | Envelope 230-252 nm in diameter, core 169-178 nm in diameter. | Observed in the sponge mesohyl. This VLP was characteristic of the Poxviridae morphology. |
| M-XLIX | GBR | *Carteriospongia foliascens* | Beaded filamentous VLP. | 340 nm long, with each bead measuring 30-35 nm in diameter. | Observed in sponge mesohyl. |
| M-L | RS | *Hyrtios erectus* | Beaded filamentous VLP. | 80-350 nm long. 15-23 nm wide. Composed of 2-8 aligned beads with 36-42 nm in longest diameter. | Observed inside vacuole of a sponge archaeocyte and in the sponge mesohyl as either a free VLP or attached to extracellular vacuole membranes. |
